# Influence of genetic polymorphism and trauma on cortical structures and PTSD severity: Imaging genetics generalized structured component analysis

**DOI:** 10.3389/fpsyt.2025.1613322

**Published:** 2025-09-17

**Authors:** Min Jin Jin, Gyeongcheol Cho, Hyeonjin Jeon, Younyoung Choi, Heungsun Hwang, Seung-Hwan Lee

**Affiliations:** ^1^ Division of Liberal Arts, Kongju National University, Gongju, Republic of Korea; ^2^ Department of Psychology, The Ohio State University, Columbus, OH, United States; ^3^ Clinical Emotion and Cognition Research Laboratory, Inje University, Goyang, Republic of Korea; ^4^ Department of Psychology, Ajou University, Suwon, Republic of Korea; ^5^ Department of Psychology, McGill University, Montreal, QC, Canada; ^6^ Institute for Hope Research, Sogang University, Seoul, Republic of Korea; ^7^ Department of Psychiatry, Inje University, Ilsan Paik Hospital, Goyang, Republic of Korea

**Keywords:** PTSD (posttraumatic stress disorder), genetic polymorphisms, brain structures, potentially traumatic events (PTEs), imaging genetics generalized structured component analysis (IG-GSCA), HTR3A, NR3C1

## Abstract

**Objective:**

The changes in brain structures affected by potentially traumatic events (PTEs) and polymorphisms of various genes are associated with posttraumatic stress disorder (PTSD). Our study investigated the pathophysiology of PTSD along with PTEs, genes, and brain regions of interest (ROIs) via imaging genetics generalized structured component analysis (IG-GSCA).

**Methods:**

A total of 231 participants (137 healthy volunteers and 94 PTSD patients) were enrolled. We performed T1-weighted structural magnetic resonance imaging, genotyping for nine genes (SLC6A4, FKBP5, ADCYAP1R1, BDNF, COMT, HTR3A, DRD2, NR3C1, and OXTR), and psychological assessments measuring PTEs, PTSD symptoms, and alcohol problems. Genes, PTEs, and their interactions were set as predictors for volumes of 60 brain ROIs, and volumes of the 60 ROIs were set as predictors for the PTSD severity, implying that volumes of brain ROIs were set to mediate the effects of genes and PTEs on the PTSD severity.

**Results:**

Our results suggested that HTR3A was related to the volume of the anterior cingulate gyrus and NR3C1 was related to the volume of the central operculum. Also, volumes of the central operculum, occipital fusiform gyrus, and anterior cingulate gyrus were negatively associated with the severity of PTSD, while PTEs were positively associated with PTSD severity.

**Conclusions:**

This study is one of the few that examined the relationships between various factors related to PTSD symptom severity, including genetics, environment, gene-environment interactions, and brain regions of interest (ROIs), all within a single model. The findings indicated mediating pathways from the HTR3A gene polymorphism to PTSD symptom severity through the volume of the anterior cingulate gyrus, and from the NR3C1 gene polymorphism to PTSD symptom severity via the volume of the central operculum. However, only the indirect effect involving NR3C1 was statistically significant. Additionally, the study found a significant association between the occipital fusiform gyrus and PTSD symptom severity.

## Introduction

Researchers studying mental illness have focused on discovering the pathological mechanisms or biological pathways that could lead to the onset of a mental illness. A recent study reported that differences in the brain seem to be associated with the general risk for mental illness, and detecting these brain differences could help clinicians evaluate an individual’s general risk for developing a mental illness (1). In particular, there is abundant evidence for decreased brain volume associated with posttraumatic stress disorder (PTSD), including the amygdala (2), insula (3, 4), anterior cingulate cortex (5), medial prefrontal cortex (4), and hippocampus (6).

People may develop PTSD after experiencing potentially traumatic events (PTEs) (7). Since PTEs may occur at different developmental stages of life, including the early childhood period in which brain development could happen, it is inevitable that those PTEs will presumably have differential impacts on the brain changes as well as the psychological traits of an individual (8). This could explain why not all individuals exposed to PTEs were diagnosed with lifetime PTSD; in a United States sample of 2,953 respondents, 82.7% were exposed to PTEs, whereas only 8.3% of those exposed to the trauma were diagnosed with lifetime PTSD (9). Therefore, the brain’s change might be a significant mediator between PTEs and PTSD symptoms. Notably, recent theoretical models propose that PTSD symptoms, such as hypervigilance, intrusive recollection, and exaggerated startle, may have evolved as adaptive responses to extreme threat, enhancing immediate survival but becoming maladaptive when prolonged or context-inappropriate (10, 11).

The change of brain structures seems to mediate not only the effects of environmental factors (e.g., PTEs) but also those of genes and their interactions on PTSD. Recent studies (12, 13) suggest that the change of brain structures could mediate the structural pathways from genes (14, 15) and environments (16, 17) to psychiatric disorders, including PTSD. For example, genetic predisposition and early environmental insults affect brain changes, which later may influence the onset of schizophrenia (18) and bipolar disorder (19, 20). Also, one study revealed that childhood PTEs and brain-derived neurotrophic factor (BDNF) polymorphism interacted to affect the cortical thickness of the left fusiform gyrus and the left transverse temporal, which in turn, were negatively associated with PTSD symptoms (21).

The gene–environment (G×E) framework has become central to contemporary PTSD research, particularly as investigations into the role of genetics and epigenetics in PTSD pathophysiology have advanced. Two recent comprehensive reviews (22, 23) synthesize findings from genome‐wide and candidate‐gene studies, highlighting nine loci that consistently demonstrate replication across independent cohorts and functional relevance to stress‐response pathways. In addition to those comprehensive review studies, PTSD is found to be associated with many genes and their genotypes (21, 24), including serotonin transporter gene (SLC6A4) (25, 26), FK506 binding protein 51 (FKBP5) (27, 28), adenylate-cyclase activating polypeptide 1 (ADCYAP1R1) (29, 30), brain-derived neurotrophic factor (BDNF) (31, 32), catechol-O-methyltransferase gene (COMT) (33, 34), 5-hydroxytryptamine receptor 3A (HTR3A) (35, 36), dopamine D2 receptor (DRD2) (37, 38), nuclear receptor subfamily 3 group C member 1 (NR3C1) (39, 40), and the oxytocin receptor gene (OXTR) (41, 42). Although genetic predispositions are expected to affect volumetric changes in the brain, leading to changes in PTSD symptoms, few studies have investigated which brain regions mediate the effects of genes on PTSD symptoms. Similarly, there is a lack of studies examining the brain regions that could mediate the pathways from environmental factors to PTSD symptoms.

Taken together, there are many individual findings about the relationships between PTEs, genes, decreased brain volume, and PTSD symptoms. To our knowledge, however, no studies have examined the pathophysiology of PTSD in a single model that encompasses all of those antecedent variables for PTSD. Therefore, this study aimed to specify and test the relationships among various genes, environmental factors, decreased volume of brain regions of interest (ROIs), and PTSD symptoms in an integrative manner via imaging genetics generalized structured component analysis (IG-GSCA) (12, 43) that could influence brain volumes simultaneously for explaining the variations of PTSD.

## Methods

### Participants

A total of 255 Korean volunteers were included in this study. Twenty-four participants were excluded due to missing values for some psychological measures and brain images, which left a final sample of 231. Healthy participants numbered 137 (59.3%), recruited from community advertisements, and patients with PTSD numbered 94 (40.7%) and recruited from notices on the bulletin board in the hospital. The PTSD patients reported their traumatic events as follows: 63 (67.02%) severe motor vehicle accidents, 9 (9.57%) defections from North Korea, 8 (8.51%) physical or sexual violence, 8 (8.51%) relationship issues, 5 (5.32%) death of a family member, and 1 (1.06%) fire. We mixed PTSD patients and healthy participants with PTE experiences in the final sample to make data on PTEs and PTSD symptoms normally distributed and to investigate the phenomenon at the level of the general population.

The total participants consisted of 75 (32.5%) men and 156 (67.5%) women, with a mean age of 46.13 years (standard deviation, *SD* = 13.51). The mean years of education were 13.04 (*SD* = 3.27). Each participant signed a written informed consent form before participating in the study. All the experimental protocols were approved by the Institutional Review Board at Inje University Ilsan Paik Hospital (IRB no. 2015 - 07-025). All measurements and experiments were carried out in accordance with the guidelines and regulations of the board and the Declaration of Helsinki.

### Psychological measures

The Korean-validated version of the *Life Events Checklist (LEC)* was used to assess the experience of PTEs (44). The LEC comprises 17 items about PTEs, and the responses include experiencing, witnessing, and learning about a PTE. This study analyzed responses to experiencing PTE, since recalling witnessing or learning about it throughout their lifetime seemed difficult and confusing to some respondents. For each of the 17 items, responses were coded as 1 if the event was experienced and 0 if not, and the sum of these scores was used in subsequent analyses.

To examine the severity of PTSD symptoms, we administered the Clinician-Administered PTSD Scale for DSM - 5 (CAPS - 5) (45). It consists of 30 items and provides information about the frequency and severity of PTSD symptoms. The severity of PTSD symptoms is rated from 0 (“absent”) to 4 (“extreme/incapacitating”). The total severity score was obtained by adding up all the item scores for each individual. A licensed psychiatrist measured PTEs using LEC and PTSD symptom severity using CAPS through a 1:1 interview, and licensed nurses or a clinical psychologist observed the entire process to secure reliability.

To adjust for the possible confounding effect of alcohol problems from other variables, the Alcohol Use Disorders Identification Test (AUDIT) was used to assess alcohol consumption, drinking behaviors, and alcohol-related problems as a covariate. The AUDIT is a 10-item screening tool developed by the World Health Organization and is well-validated in the Korean population (46). The AUDIT is assessed on a 5-point Likert scale ranging from 0 (“never”) to 4 (“4 or more times a week”).

### DNA genotyping

We used the same DNA genotyping process as in previous studies (12, 21). Participants had their blood sampled to extract DNA using a NanoDrop^®^ ND - 1000 UV-Vis Spectrophotometer. Then, genomic DNA was diluted to a 5 ng/µl concentration on 96-well polymerase chain reaction (PCR) plates. TaqMan SNP genotyping assays were obtained from Applied Biosystems. Afterward, the probes were labeled with either the FAM or the VIC dye at the 5’ end and with a minor-groove binder and a non-fluorescent quencher at the 3’ end. PCR was performed in 5 μl of the mixture containing 2 μl of a DNA sample, 0.125 μl of each TaqMan™ SNP Genotyping Assay (Thermo Fisher Scientific, USA), 2.5 μl of the TaqMan™ Genotyping Master Mix (Thermo Fisher Scientific, USA), and 0.375 μl of distilled water. Amplification and detection were carried out with a detection system (QuantStudio 12K Flex Real-Time PCR System, Thermo Fisher Scientific, USA), using the following profile: 50 °C for 2 min, 95 °C for 10 min, followed by 60 cycles of 95 °C for 15 sec, and 60 °C for 1 min. After the PCR amplification, allelic discrimination (an endpoint plate read) was performed on the same machines (the QuantStudio 12K Flex Real-Time PCR System). The QuantStudio 12K Flex Software calculated the fluorescence measured during the plate read and plotted Rn values based on the signals coming from each well. Subsequently, automatic or manual allele calls were performed on the analyzed plates. Three positive samples and one negative control sample were present on each plate. We confirmed the clustering image with positive controls. Intra-genomic DNA (gDNA) samples of known genotypes were used for the positive control. Guided by two recent comprehensive studies (22, 23) and other multiple empirical reports, we therefore selected the following nine genes (18 SNPs) for inclusion in our IG‐GSCA model: SLC6A4, FKBP5, ADCYAP1R1, BDNF, COMT, HTR3A, DRD2, NR3C1, and OXTR. A total of nine genes with 18 SNPs were analyzed and are described in **Table 1**. For further analyses, the number of minor alleles was coded for each genotype: 0 for wild types with zero minor alleles, 1 for hetero types with one minor allele, and 2 for mutant types with two minor alleles.

**Table 1:** List of genes and their SNP indicators included in analyses.

Gene name	rs number	wild [N(%)]		hetero [N(%)]		mutant [N(%)]	
SLC6A4	rs25531	AA	174 (75.3%)	AG	57 (24.7%)	GG	0 (0%)
FKBP5	rs9296158	GG	115 (49.8%)	AG	98 (42.4%)	AA	18(7.8%)
	rs3800373	AA	148 (64.1%)	AC	71 (30.7%)	CC	12 (5.2%)
	rs1360780	CC	144 (62.3%)	CT	74 (32.0%)	TT	13 (5.6%)
	rs9470080	CC	111 (48.1%)	CT	100 (43.2%)	TT	20 (8.7%)
	rs4713916	GG	146 (63.2%)	AG	74 (32.0%)	AA	74 (32.0%)
	rs4713919	GG	130 (56.3%)	AG	82 (35.5%)	AA	19 (8.2%)
	rs6902321	TT	118 (51.1%)	CT	97 (42.0%)	CC	16 (6.9%)
	rs56311918	TT	168 (72.7%)	CT	59 (25.5%)	CC	4 (1.7%)
	rs3798345	CC	157 (68.0%)	CT	66 (28.5%)	TT	8 (3.5%)
ADCYAP1R1	rs2267735	CC	59 (25.5%)	CG	119 (51.5%)	GG	53 (22.9%)
BDNF	rs6265	CC	75 (32.5%)	CT	108 (46.8%)	TT	48 (20.8%)
COMT	rs4680	GG	116 (50.2%)	AG	102 (44.2%)	AA	13 (5.6%)
	rs4633	CC	119 (51.5%)	CT	100 (43.3%)	TT	12 (5.2%)
HTR3A	rs1062613	CC	194 (84.0%)	CT	34 (14.7%)	TT	3 (1.3%)
DRD2	rs2075652	GG	86 (37.2%)	GA	99 (42.9%)	AA	46 (19.9%)
NR3C1	rs258747	AA	125 (54.1%)	AG	89 (38.5%)	GG	17 (7.4%)
OXTR	rs53576	AA	92 (39.8%)	AG	99 (42.9%)	GG	40 (17.3%)

SLC6A4, serotonin transporter gene; FKBP5, FK506 binding protein 51; ADCYAP1R1, adenylate-cyclase activating polypeptide 1; BDNF, brain-derived neurotrophic factor; COMT, catechol-O-methyltransferase gene; HTR3A, 5-hydroxytryptamine receptor 3A; DRD2, dopamine D2 receptor; NR3C1, nuclear receptor subfamily 3 group C member 1; OXTR, oxytocin receptor gene.

### MRI acquisition and processing and voxel-based morphometry

MRI was performed using a 1.5 T scanner (Magneton Avanto, Siemens, Erlangen, Germany). Head motion was minimized with restraining foam pads provided by the manufacturer. High-resolution T1-weighted MRI images were acquired with the acquisition parameters of a 227 × 384 acquisition matrix, a 210 × 250 field-of-view, 0.9 × 0.7 × 1.2 voxel size, a total of 87,168 voxels, a TE of 3.42 ms, a TR of 1,900 ms, 1.2-mm slice thickness, and a flip angle of 15°.

We used the same MRI process as the one used in the previous study (47). Images were inspected visually for motion or other artifacts before and after preprocessing. The voxel-based volumetry (VBM) was conducted using CAT12 (http://dbm.neuro.uni-jena.de/cat/) implemented in SPM12 (Wellcome Department of Cognitive Neurology, London, UK). SPM12 tissue probability maps were used for the initial spatial registration. The structural T1 images were regularized with an ICBM East Asian template and normalized using the DARTEL algorithm (48). The images were then segmented into gray matter, white matter, and cerebrospinal fluid (49). Jacobian-transformed tissue probability maps were used to modulate images. The volume of the brain parts was extracted using the Neuromorphometric atlas (http://neuromorphometrics.com/) from 142 parts from 67 ROIs. Divided by a subject’s total brain volume, the extracted volumes of the brain parts were transformed into the relative sizes of volumes to adjust for individual differences in overall brain volume (50). Among the 67 ROIs in total, we excluded seven subcortical ROIs that were far from cortical function related to PTSD; cerebral exterior for it is considered residual non‐brain tissue from skull‐stripping (51), cerebellar vermis, brain stem, and optic chiasm since they have not shown consistent volumetric abnormalities in PTSD voxel‐based morphometry meta‐analyses (52), cerebellar and cerebral white matter for they are conceptually distinct from the gray‐matter volumetric framework (53), and cerebrospinal fluid since it is considered as an indirect marker of brain atrophy rather than as a mediator in structural models (54). A total of 60 remaining ROIs as potential mediators for the pathways from the nine genes to PTSD in the model. The list of the 60 ROIs and the means and SDs of their volumes are provided in **Table 2**.

**Table 2:** Mean and standard deviation of volumes (cm^3^) of ROIs and their indicators included in the analysis.

ROIs	Indicator	Mean ± SD	ROIs	Indicator	Mean ± SD
Frontal Pole (FRP)	Left-	2.716 ± 0.371	Supramarginal Gyrus (SMG)	Left-	8.042 ± 1.096
	Right-	3.140 ± 0.464		Right-	7.123 ± 0.977
Superior Frontal Gyrus (SFG)	Left-	12.913 ± 1.830	Superior Parietal Lobule (SPL)	Left-	9.438 ± 1.236
	Right-	12.802 ± 1.786		Right-	9.019 ± 1.253
Middle Frontal Gyrus (MFG)	Left-	17.537 ± 2.510	Angular Gyrus (AnG)	Left-	8.743 ± 1.171
	Right-	17.361 ± 2.590		Right-	10.186 ± 1.344
Inferior Frontal Gyrus (IFG)	Left opercular-	3.168 ± 0.534	Postcentral Gyrus (MPoG, medial segment)	Left-	0.800 ± 0.162
	Right opercular-	3.279 ± 0.511		Right-	0.843 ± 0.190
	Left orbital-	1.301 ± 0.232	Precuneus (PCu)	Left-	10.035 ± 1.411
	Right orbital-	1.310 ± 0.226		Right-	10.491 ± 1.469
	Left triangular-	2.996 ± 0.479	Superior Occipital Gyrus (SOG)	Left-	2.944 ± 0.448
	Right triangular-	3.093 ± 0.495		Right-	3.645 ± 0.543
Precentral Gyrus (PrG)	Left-	5.739 ± 0.824	Inferior Occipital Gyrus (IOG)	Left-	5.666 ± 0.844
	Right-	7.026 ± 1.079		Right-	5.932 ± 0.903
Superior Frontal Gyrus (MSFG; medial segment)	Left-	10.146 ± 1.470	Middle Occipital Gyrus (MOG)	Left-	5.637 ± 0.792
	Right-	10.109 ± 1.508		Right-	4.494 ± 0.651
Supplementary Motor Cortex (SMC)	Left-	5.711 ± 0.751	Occipital Pole	Left-	2.826 ± 0.534
	Right-	5.721 ± 0.729		Right-	2.494 ± 0.524
Medial Frontal Cortex (MFC)	Left-	1.722 ± 0.288	Occipital Fusiform Gyrus (OFuG)	Left-	3.157 ± 0.492
	Right-	1.878 ± 0.334		Right-	3.099 ± 0.493
Gyrus Rectus (Gre)	Left-	1.933 ± 0.279	Cuneus (Cun)	Left-	3.607 ± 0.649
	Right-	1.884 ± 0.291		Right-	4.135 ± 0.725
Subcallosal Area (SCA)	Left-	1.171 ± 0.175	Calcarine Cortex (Calc)	Left-	3.091 ± 0.642
	Right-	1.192 ± 0.177		Right-	3.251 ± 0.645
Precentral Gyrus (MPrG; medial segment)	Left-	2.000 ± 0.339	Lingual Gyrus (LiG)	Left-	6.470 ± 0.902
	Right-	2.079 ± 0.357		Right-	7.041 ± 1.071
Anterior Orbital Gyrus (AOrG)	Left-	1.882 ± 0.268	Anterior cingulate gyrus (ACgG)	Left-	5.032 ± 0.778
	Right-	1.940 ± 0.322		Right-	3.813 ± 0.662
Medial Orbital Gyrus (MOrG)	Left-	4.136 ± 0.534	Middle cingulate gyrus (MCgG)	Left-	4.195 ± 0.626
	Right-	4.243 ± 0.618		Right-	4.400 ± 0.661
Lateral Orbital Gyrus (LOrG)	Left-	1.937 ± 0.296	Posterior cingulate gyrus (PCgG)	Left-	3.963 ± 0.593
	Right-	2.000 ± 0.352		Right-	3.712 ± 0.558
Posterior Orbital Gyrus (POrG)	Left-	2.530 ± 0.391	Parahippocampal Gyrus (PHG)	Left-	2.940 ± 0.315
	Right-	2.523 ± 0.418		Right-	2.816 ± 0.337
Frontal Operculum (FO)	Left-	1.840 ± 0.261	Entorhinal Area (Ent)	Left-	2.288 ± 0.303
	Right-	1.896 ± 0.298		Right-	2.273 ± 0.296
Central Operculum (CO)	Left-	3.975 ± 0.594	Third Ventricle	Left-	0.043 ± 0.010
	Right-	3.888 ± 0.584		Right-	0.036 ± 0.009
Parietal Operculum (PO)	Left-	2.362 ± 0.433	Fourth Ventricle	Left-	0.060 ± 0.011
	Right-	2.028 ± 0.397		Right-	0.062 ± 0.012
Anterior Insula (AIns)	Left-	4.380 ± 0.554	Inferior Lateral Ventricles	Right-	0.008 ± 0.003
	Right-	4.389 ± 0.562	Lateral Ventricles	Left-	0.587 ± 0.169
Posterior Insula (PIns)	Left-	2.161 ± 0.268		Right-	0.417 ± 0.127
	Right-	2.456 ± 0.328	Caudate	Left-	2.850 ± 0.404
Temporal Pole (TMP)	Left-	8.565 ± 1.159		Right-	2.856 ± 0.426
	Right-	8.684 ± 1.318	Putamen	Left-	3.441 ± 0.541
Superior Temporal Gyrus (STG)	Left-	6.132 ± 0.864		Right-	3.358 ± 0.544
	Right-	6.422 ± 0.884	Thalamus	Left-	5.076 ± 0.636
Middle Temporal Gyrus (MTG)	Left-	13.885 ± 1.793		Right-	5.235 ± 0.668
	Right-	14.000 ± 1.951	Basal Forebrain	Left-	0.693 ± 0.078
Inferior Temporal Gyrus (ITG)	Left-	10.901 ± 1.372		Right-	0.699 ± 0.082
	Right-	11.384 ± 1.611	Nucleus Accumbens	Left-	0.394 ± 0.057
Planum Polare (PP)	Left-	2.093 ± 0.286		Right-	0.380 ± 0.059
	Right-	1.737 ± 0.264	Pallidum	Left-	0.237 ± 0.065
Transverse Temporal Gyrus (TTG)	Left-	1.322 ± 0.275		Right-	0.239 ± 0.064
	Right-	1.143 ± 0.213	Ventral Diencephalon	Left-	0.789 ± 0.096
Planum Temporal (PT)	Left-	1.952 ± 0.385		Right-	0.749 ± 0.089
	Right-	1.834 ± 0.320	Amygdala	Left-	0.911 ± 0.104
Fusiform Gyrus (FuG)	Left-	7.921 ± 1.016		Right-	0.879 ± 0.113
	Right-	7.669 ± 1.125	Hippocampus	Left-	3.092 ± 0.332
Postcentral Gyrus (PoG)	Left-	8.773 ± 1.328		Right-	3.418 ± 0.413
	Right-	7.832 ± 1.196			

### Statistical analysis–IG-GSCA

We used a component-based structural equation modeling technique specifically developed for imaging genetics research, called Imaging Genetics Generalized Structured Component Analysis (IG-GSCA) (12, 43). IG-GSCA enables researchers to statistically model and test path-analytic relationships among various constructs, representing those constructs as weighted composites of observed variables—termed *components*. For instance, a gene can be viewed a genetic construct composed of multiple SNPs located within the gene (55), whereas a brain ROI can be seen as an imaging construct comprising the voxels within the ROI (56). IG-GSCA generates a component for each gene and ROI from the relevant SNPs and brain voxels, respectively, and specifies biologically plausible pathways between genes, ROIs, and behavioral phenotypes within a single modeling framework. It also accommodates various well-documented interactions between constructs (57) in imaging genetics studies, such as gene-gene interactions (epistasis; G×G) (58) or gene-environment interaction (G×E) (59). The observed genetic or imaging variables used to define are referred to as *indicators* of their respective constructs.

Unlike univariate approaches—which estimate parameters for each equation separately and thus require *post hoc* corrections (e.g., Bonferroni, Šidák, Benjamini–Hochberg) to control the family‐wise error—IG-GSCA jointly estimates all parameters within a unified multivariate model. This simultaneous estimation accounts for shared variance and inherently mitigates Type I error inflation without the need for external corrections. Moreover, it enables IG-GSCA to statistically test mediation effects, such as whether ROIs mediate the relationship between genes and behavioral phenotypes. These features make IG-GSCA especially well-suited for imaging genetics studies that examine pathways from genetic variations to changes in brain structure to behavioral outcomes (60).

Lastly, IG-GSCA incorporates regularization techniques (61) to address multicollinearity, a common issue in imaging genetics (62) For example, neighboring genes often exhibit high correlations, which may cause multicollinearity when included simultaneously as predictors for the same outcome. In general, multicollinearity leads to unstable parameter estimates with inflated standard errors, especially in studies with small sample sizes. Regularization helps prevents overfitting to sample-specific noise, yielding more stable estimates and better generalizability. Given our hypothesized model includes a large number of genetic and brain-imaging variables as well as their interaction terms —relative to a modest sample size (N = 231), we employed IG-GSCA with regularization. A simulation study using a model of comparable complexity (e.g., nine-gene components, nine G×E interactions, 60 ROI components, and one outcome) demonstrates IGSCA performs as expected with a sample size of N = 250 (12).

### Model specification

**Figure 1:**
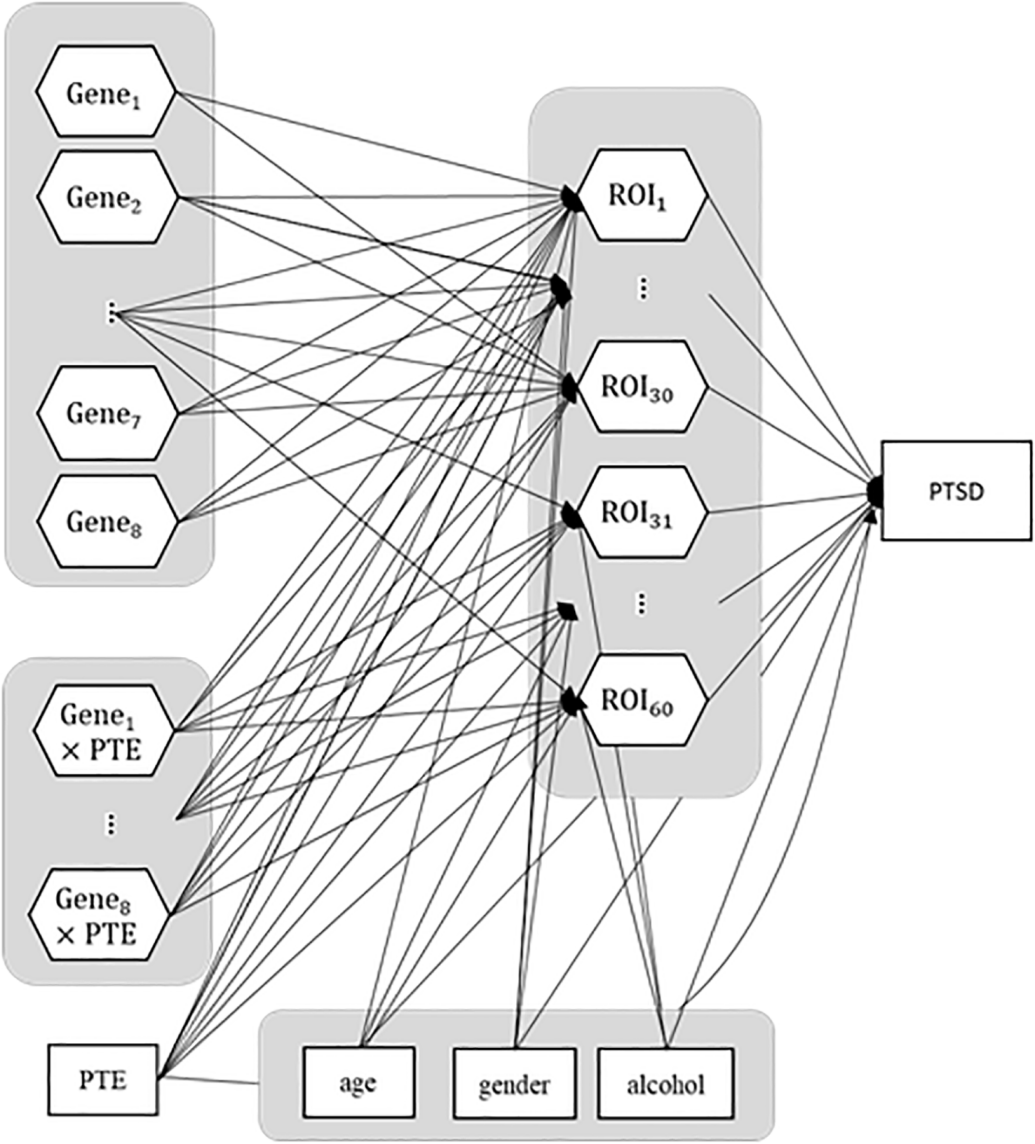
Hypothesized model specification. Hexagons represent constructs, whereas squares are observed variables. PTE, potentially traumatic event; ROI, regions of interest; PTSD, posttraumatic stress disorder.

Since recent studies suggested that the change of brain structures could mediate the pathways from genes and environmental factors to psychiatric disorders (12), we hypothesized a structural model where brain ROIs with cortical structural features mediate the effects of genes and PTEs on PTSD symptom severity. A total of nine genes with 18 SNPs were selected based on their relevance to PTSD (25, 27, 29, 31, 33, 36, 37, 39, 41). The number of minor alleles was coded for each genotype: 0 for wild types with zero minor alleles, 1 for hetero types with one minor allele, and 2 for mutant types with two minor alleles for further analyses. Also, the interaction effects of genes and PTEs were additionally considered based on the previous findings (63, 64). Among the 67 ROIs from the whole brain, we excluded seven subcortical ROIs that are far from cortical function related to PTSD (i.e., cerebral exterior, cerebellar vermis, brain stem, optic chiasm, cerebellar and cerebral white matter, and cerebrospinal fluid) from the model, leaving a total of 60 ROIs as mediating variables in the model. In addition, we set age, gender, and alcohol-related problems measured with AUDIT as covariates to control for possible contaminating effects (65–68). In all, we contemplated the structural model involving the pathways from nine genes and PTEs to 60 brain ROIs, the paths from 60 brain ROIs and PTEs to PTSD symptom severity, and the paths from three covariates (gender, age, and AUDIT score) to 60 brain ROIs and PTSD symptom severity. The total number of pathways specified in the structural model was 1,384. The structural model is displayed in **Figure 1**, in which each individual pathway between two constructs, termed a path coefficient parameter, is symbolized by an arrow between the two constructs.

## Results

### Descriptive statistics

The demographic and psychological characteristics of healthy and PTSD participants are presented in **Table 3**. The two groups did not show statistically significant gender differences (*t* = 1.642, *p* = 0.252), age difference (*t* = 1.309, *p* = 0.192), or AUDIT (*t* = -1.144, *p* = 0.254), whereas their PTE and PTSD scores were significantly different (*t* = -4.823, *p* < 0.001 for PTEs; *t* = -13.348, *p* < 0.001 for PTSD). Meanwhile, all variables, including both LEC scores and PTSD symptom severity scores, were normally distributed even for the total pool of participants since each skewness score was below 2.0 and each kurtosis score was below 7.0.

**Table 3:** Comparison of demographic, psychological, and behavioral characteristics in participants.

	Total participants (N = 231)	Healthy participants (N = 137)	PTSD participants (N = 94)	*t*	*p*
	*Mean* ± *SD* or N (%)				
Gender					
Male	75 (32.5)	40 (29.2)	35 (37.2)	1.642	.252
Female	156 (67.5)	97 (70.8)	59 (62.8)		
Age	46.10 ± 13.49	47.05 ± 13.58	44.72 ± 13.32	1.309	.192
AUDIT	3.04 ± 3.61	2.80 ± 3.43	3.38 ± 3.86	-1.144	.254
PTEs	3.72 ± 2.47	3.10 ± 2.35	4.63 ± 2.37	**-4.828**	**<.001**
PTSD symptom severity	28.20 ± 20.33	16.87 ± 13.81	44.71 ± 16.68	**-13.348**	**<.001**

*t* and *p* values show the results from pairwise sample t-tests for each variable between healthy and PTSD subjects. AUDIT, Alcohol Use Disorders Identification Test; PTE, posttraumatic events measured by Life Events Checklist (LEC).

bold values represents significant p values.

### Model analysis

We tested an IG-GSCA model in which nine candidate genes (18 SNPs), their interactions with PTEs, and three covariates (age, gender, AUDIT score) were linked to PTSD symptom severity via 60 cortical ROIs. In total, the model specified 1,384 pathways, and its structure is shown in **Figure 1**.

Each set of indicators for genes and ROIs is described in **Tables 1** and **2**. The number of SNP indicators per gene ranged from 1 to 8. Each ROI construct was composed of the corresponding ROIs on both brain hemispheres except the inferior frontal gyrus (IFG). The three sub-parts (e.g., opercular, orbital, and triangular) of IFG on each side of the brain were used as indicators of IFG, as indicated in the Neuromorphometric Atlas. The constructs for G×E interaction did not have indicators since they were defined as products of a pair of genes and PTE.

### IG-GSCA results

We fitted our hypothesized IG-GSCA model to data via GSCA Pro (69). We adopted ridge-regularization for the structural part of our model and considered values between 0 and 1,000 as candidate values of the ridge tuning parameter λ. We selected λ = 62 based on 10 × 5 fold cross-validation. We employed 5,000 bootstrap samples to estimate standard errors and 95% confidence intervals for the parameter estimates.

We calculated the values of three goodness-of-fit measures: FIT, the goodness-of-fit index (GFI), and the standardized root mean square residual (SRMR). FIT is computed based on the residuals for components and indicators, whereas GFI and SRMR are calculated based on the discrepancy between the sample and reproduced covariance matrices of indicators. A higher value of FIT or GFI indicates a better fit, whereas a lower value of SRMR suggests a better fit. IG-GSCA provided the FIT value of 0.640, indicating that the model explained 64.0% of the total variance of all the components and indicators in the model. It also yielded a GFI value of 0.989, which is far greater than the recommended cutoff criterion (0.93) (70). Lastly, it produced the SRMR value of 0.04, which is much smaller than the recommended cutoff criterion (0.08) (70).

**Table 4** presents standardized loading estimates, which can be interpreted as the correlations between constructs and their indicators. All the loading estimates were statistically significant, and their effect sizes were large (i.e., at least larger than 0.7), indicating that components were constructed to well explain the variations of their indicators. The average R^2^ for all the indicators was 0.87.

**Table 4:** The estimates of loadings and their standard errors and 95% confidence intervals.

Name		Loadings			
Constructs	Indicator	Estimate	SE	95% CI	
*SLC6A4*	rs25531	1.00	0.00	1.00	1.00
*FKBP5*	rs9296158	0.89	0.02	0.85	0.92
	rs3800373	0.89	0.03	0.83	0.93
	rs1360780	0.92	0.01	0.89	0.95
	rs9470080	0.91	0.02	0.88	0.94
	rs4713916	0.92	0.01	0.90	0.95
	rs4713919	0.85	0.02	0.80	0.89
	rs6902321	0.89	0.02	0.85	0.92
	rs56311918	0.83	0.03	0.78	0.88
	rs3798345	0.89	0.02	0.84	0.93
*ADCYAP1R1*	rs2267735	1.00	0.00	1.00	1.00
*BDNF*	rs6265	1.00	0.00	1.00	1.00
*COMT*	rs4680	0.96	0.03	0.89	0.99
	rs4633	1.00	0.01	0.97	1.00
*HTR3A*	rs1062613	1.00	0.00	1.00	1.00
*DRD2*	rs2075652	1.00	0.00	1.00	1.00
*NR3C1*	rs258747	1.00	0.00	1.00	1.00
*OXTR*	rs53576	1.00	0.00	1.00	1.00
PTE	post traumatic events	1.00	0.00	1.00	1.00
Frontal Pole (FRP)	Left FRP frontal pole	0.92	0.02	0.88	0.94
	Right FRP frontal pole	0.90	0.01	0.87	0.93
Superior Frontal Gyrus (SFG)	Left SFG superior frontal gyrus	0.96	0.01	0.95	0.97
	Right SFG superior frontal gyrus	0.95	0.01	0.94	0.96
Middle Frontal Gyrus (MFG)	Left MFG middle frontal gyrus	0.97	0.00	0.96	0.98
	Right MFG middle frontal gyrus	0.96	0.01	0.95	0.97
Inferior Frontal Gyrus (IFG)	Left OpIFG opercular part of the inferior frontal gyrus	0.73	0.04	0.65	0.80
	Right OpIFG opercular part of the inferior frontal gyrus	0.75	0.03	0.68	0.81
	Left OrIFG orbital part of the inferior frontal gyrus	0.75	0.03	0.68	0.81
	Right OrIFG orbital part of the inferior frontal gyrus	0.80	0.03	0.74	0.84
	Left TrIFG triangular part of the inferior frontal gyrus	0.83	0.02	0.77	0.87
	Right TrIFG triangular part of the inferior frontal gyrus	0.83	0.02	0.78	0.87
Precentral Gyrus (PrG)	Left PrG precentral gyrus	0.96	0.01	0.94	0.97
	Right PrG precentral gyrus	0.96	0.01	0.94	0.97
Superior Frontal Gyrus (MSFG; medial segment)	Left MSFG superior frontal gyrus medial segment	0.96	0.01	0.95	0.97
	Right MSFG superior frontal gyrus medial segment	0.95	0.01	0.94	0.96
Supplementary Motor Cortex (SMC)	Left SMC supplementary motor cortex	0.96	0.01	0.94	0.97
	Right SMC supplementary motor cortex	0.96	0.01	0.95	0.97
Medial Frontal Cortex (MFC)	Left MFC medial frontal cortex	0.95	0.01	0.93	0.96
	Right MFC medial frontal cortex	0.94	0.01	0.92	0.96
Gyrus Rectus (Gre)	Left GRe gyrus rectus	0.94	0.01	0.92	0.95
	Right GRe gyrus rectus	0.94	0.01	0.92	0.96
Subcallosal Area (SCA)	Left SCA subcallosal area	0.91	0.01	0.88	0.93
	Right SCA subcallosal area	0.90	0.01	0.88	0.93
Precentral Gyrus (MPrG; medial segment)	Left MPrG precentral gyrus medial segment	0.94	0.01	0.92	0.95
	Right MPrG precentral gyrus medial segment	0.94	0.01	0.93	0.96
Anterior Orbital Gyrus (AOrG)	Left AOrG anterior orbital gyrus	0.91	0.01	0.89	0.93
	Right AOrG anterior orbital gyrus	0.90	0.01	0.87	0.92
Medial Orbital Gyrus (MOrG)	Left MOrG medial orbital gyrus	0.95	0.01	0.93	0.96
	Right MOrG medial orbital gyrus	0.92	0.01	0.90	0.95
Lateral Orbital Gyrus (LOrG)	Left LOrG lateral orbital gyrus	0.93	0.01	0.90	0.95
	Right LOrG lateral orbital gyrus	0.92	0.01	0.90	0.94
Posterior Orbital Gyrus (POrG)	Left POrG posterior orbital gyrus	0.94	0.01	0.92	0.96
	Right POrG posterior orbital gyrus	0.94	0.01	0.91	0.96
Frontal Operculum (FO)	Left FO frontal operculum	0.92	0.01	0.89	0.94
	Right FO frontal operculum	0.92	0.01	0.90	0.94
Central Operculum (CO)	Left CO central operculum	0.94	0.01	0.91	0.95
	Right CO central operculum	0.94	0.01	0.92	0.95
Parietal Operculum (PO)	Left PO parietal operculum	0.92	0.01	0.90	0.94
	Right PO parietal operculum	0.92	0.01	0.89	0.94
Anterior Insula (AIns)	Left AIns anterior insula	0.97	0.01	0.96	0.98
	Right AIns anterior insula	0.97	0.01	0.96	0.99
Posterior Insula (PIns)	Left PIns posterior insula	0.96	0.01	0.95	0.97
	Right PIns posterior insula	0.96	0.01	0.94	0.97
Temporal Pole (TMP)	Left TMP temporal pole	0.92	0.03	0.86	0.96
	Right TMP temporal pole	0.92	0.03	0.87	0.96
Superior Temporal Gyrus (STG)	Left STG superior temporal gyrus	0.89	0.02	0.86	0.92
	Right STG superior temporal gyrus	0.90	0.01	0.87	0.93
Middle Temporal Gyrus (MTG)	Left MTG middle temporal gyrus	0.92	0.01	0.90	0.94
	Right MTG middle temporal gyrus	0.95	0.01	0.92	0.97
Inferior Temporal Gyrus (ITG)	Left ITG inferior temporal gyrus	0.90	0.02	0.86	0.93
	Right ITG inferior temporal gyrus	0.90	0.02	0.86	0.95
Planum Polare (PP)	Left PP planum polare	0.94	0.01	0.93	0.96
	Right PP planum polare	0.94	0.01	0.91	0.96
Transverse Temporal Gyrus (TTG)	Left TTG transverse temporal gyrus	0.91	0.01	0.88	0.94
	Right TTG transverse temporal gyrus	0.93	0.01	0.90	0.95
Planum Temporal (PT)	Left PT planum temporale	0.89	0.02	0.86	0.92
	Right PT planum temporale	0.90	0.01	0.86	0.92
Fusiform Gyrus (FuG)	Left FuG fusiform gyrus	0.93	0.02	0.89	0.95
	Right FuG fusiform gyrus	0.92	0.03	0.88	0.97
Postcentral Gyrus (PoG)	Left PoG postcentral gyrus	0.95	0.01	0.93	0.96
	Right PoG postcentral gyrus	0.94	0.01	0.92	0.96
Supramarginal Gyrus (SMG)	Left SMG supramarginal gyrus	0.92	0.01	0.90	0.94
	Right SMG supramarginal gyrus	0.93	0.01	0.91	0.95
Superior Parietal Lobule (SPL)	Left SPL superior parietal lobule	0.93	0.01	0.91	0.95
	Right SPL superior parietal lobule	0.94	0.01	0.91	0.95
Angular Gyrus (AnG)	Left AnG angular gyrus	0.91	0.01	0.88	0.93
	Right AnG angular gyrus	0.94	0.01	0.92	0.95
Postcentral Gyrus (MPoG, medial segment)	Left MPoG postcentral gyrus medial segment	0.86	0.02	0.82	0.89
	Right MPoG postcentral gyrus medial segment	0.85	0.02	0.81	0.89
Precuneus (PCu)	Left PCu precuneus	0.97	0.00	0.96	0.98
	Right PCu precuneus	0.97	0.00	0.96	0.98
Superior Occipital Gyrus (SOG)	Left SOG superior occipital gyrus	0.89	0.02	0.86	0.92
	Right SOG superior occipital gyrus	0.89	0.02	0.86	0.92
Inferior Occipital Gyrus (IOG)	Left IOG inferior occipital gyrus	0.89	0.02	0.86	0.92
	Right IOG inferior occipital gyrus	0.90	0.02	0.87	0.93
Middle Occipital Gyrus (MOG)	Left MOG middle occipital gyrus	0.89	0.01	0.86	0.91
	Right MOG middle occipital gyrus	0.91	0.01	0.88	0.93
Occipital Pole	Left OCP occipital pole	0.86	0.02	0.82	0.90
	Right OCP occipital pole	0.85	0.02	0.80	0.88
Occipital Fusiform Gyrus (OFuG)	Left OFuG occipital fusiform gyrus	0.86	0.02	0.81	0.89
	Right OFuG occipital fusiform gyrus	0.89	0.02	0.86	0.92
Cuneus (Cun)	Left Cun cuneus	0.94	0.01	0.92	0.96
	Right Cun cuneus	0.94	0.01	0.92	0.96
Calcarine Cortex (Calc)	Left Calc calcarine cortex	0.96	0.01	0.95	0.97
	Right Calc calcarine cortex	0.96	0.01	0.95	0.97
Lingual Gyrus (LiG)	Left LiG lingual gyrus	0.92	0.01	0.89	0.95
	Right LiG lingual gyrus	0.91	0.03	0.87	0.96
Anterior cingulate gyrus (ACgG)	Left ACgG anterior cingulate gyrus	0.94	0.01	0.92	0.95
	Right ACgG anterior cingulate gyrus	0.91	0.01	0.89	0.93
Middle cingulate gyrus (MCgG)	Left MCgG middle cingulate gyrus	0.94	0.01	0.92	0.95
	Right MCgG middle cingulate gyrus	0.94	0.01	0.92	0.96
Posterior cingulate gyrus (PCgG)	Left PCgG posterior cingulate gyrus	0.96	0.01	0.94	0.97
	Right PCgG posterior cingulate gyrus	0.95	0.01	0.93	0.96
Parahippocampal Gyrus (PHG)	Left PHG parahippocampal gyrus	0.93	0.02	0.89	0.95
	Right PHG parahippocampal gyrus	0.94	0.02	0.90	0.96
Entorhinal Area (Ent)	Left Ent entorhinal area	0.92	0.02	0.88	0.95
	Right Ent entorhinal area	0.93	0.01	0.89	0.95
Third Ventricle	Left 3rd Ventricle	0.96	0.01	0.95	0.97
	Right 3rd Ventricle	0.96	0.01	0.95	0.97
Fourth Ventricle	Left 4th Ventricle	0.96	0.01	0.94	0.97
	Right 4th Ventricle	0.95	0.01	0.93	0.97
Inferior Lateral Ventricles	Right Inf Lat Vent	1.00	0.00	1.00	1.00
Lateral Ventricles	Left Lateral Ventricle	0.92	0.02	0.88	0.94
	Right Lateral Ventricle	0.92	0.01	0.89	0.94
Caudate	Left Caudate	0.98	0.00	0.97	0.99
	Right Caudate	0.98	0.00	0.98	0.99
Putamen	Left Putamen	0.99	0.00	0.98	0.99
	Right Putamen	0.97	0.01	0.96	0.98
Thalamus	Left Thalamus Proper	0.98	0.01	0.96	0.99
	Right Thalamus Proper	0.98	0.01	0.96	0.99
Basal Forebrain	Left Basal Forebrain	0.95	0.01	0.93	0.96
	Right Basal Forebrain	0.95	0.01	0.93	0.96
Nucleus Accumbens	Right Accumbens Area	0.97	0.01	0.95	0.98
	Left Accumbens Area	0.98	0.01	0.96	0.99
Pallidum	Left Pallidum	0.96	0.01	0.94	0.97
	Right Pallidum	0.96	0.01	0.94	0.98
Ventral Diencephalon	Left Ventral DC	0.97	0.01	0.96	0.98
	Right Ventral DC	0.95	0.01	0.94	0.97
Amygdala	Right Amygdala	0.94	0.01	0.92	0.97
	Left Amygdala	0.94	0.02	0.92	0.97
Hippocampus	Left Hippocampus	0.94	0.02	0.90	0.98
	Right Hippocampus	0.93	0.02	0.90	0.97
PTSD	CAPS-5 severity	1.00	0.00	1.00	1.00
gender	gender	1.00	0.00	1.00	1.00
age	age	1.00	0.00	1.00	1.00
AUDIT	AUDIT	1.00	0.00	1.00	1.00

SLC6A4, serotonin transporter gene; FKBP5, FK506 binding protein 51; ADCYAP1R1, adenylate-cyclase activating polypeptide 1; BDNF, brain-derived neurotrophic factor; COMT, catechol-O-methyltransferase gene; HTR3A, 5-hydroxytryptamine receptor 3A; DRD2, dopamine D2 receptor; NR3C1, nuclear receptor subfamily 3 group C member 1; OXTR, oxytocin receptor gene; PTE, potentially traumatic event; PTSD, posttraumatic stress disorder.

**Figure 2:**
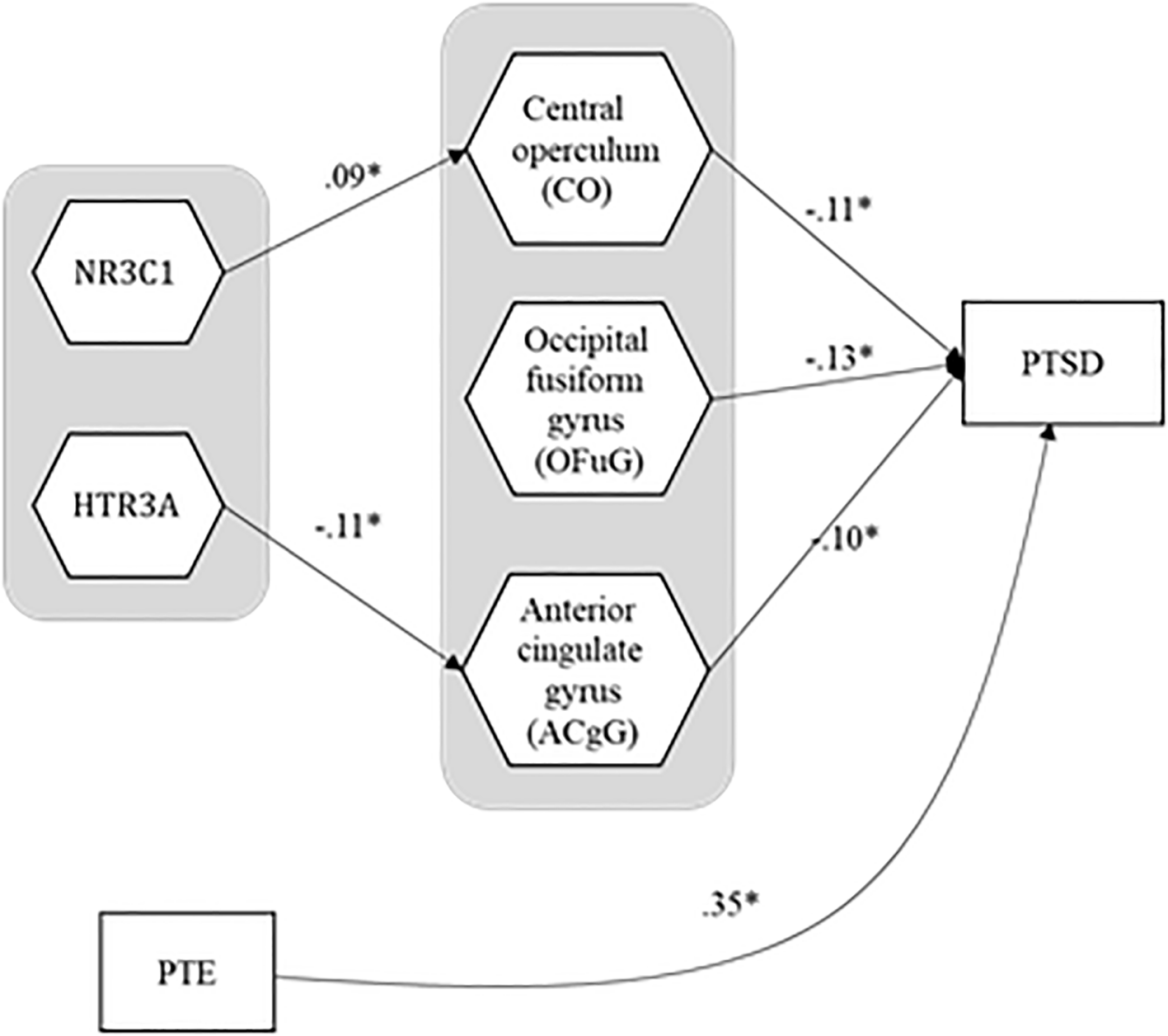
The estimates for the path coefficients that form statistically significant linkages from genes and PTE to ROIs to PTSD symptom severity. PTE, potentially traumatic event; PTSD, posttraumatic stress disorder.

Among 187 significant path coefficients, we paid most attention to reporting and interpreting the statistically significant path coefficient estimates from ROIs to the severity of PTSD symptoms and the ones from genes or PTEs to ROIs, since this study aimed to identify the mediating pathways from genes or PTEs to ROIs to the severity of PTSD symptoms. **Figure 2** presents the mediating model, and **Table 5** provides the standardized estimates for those path coefficients. The estimates of the entire path coefficients are presented in **Supplementary Material**.

**Table 5:** The estimates for the path coefficients that form statistically significant linkages from genes and PTE to ROIs to PTSD symptom severity, and their standard errors and 95% confidence intervals (direct effects only).

Path	Estimate	SE	95% CI	
HTR3A(gene) → Anterior cingulate gyrus (ACgG)	-0.11	0.04	-0.18	-0.03
NR3C1(gene) → Central operculum (CO)	0.09	0.03	0.02	0.15
Central operculum (CO) → PTSD symptom severity	-0.11	0.04	-0.18	-0.00
Occipital fusiform gyrus (OFuG) → PTSD symptom severity	-0.13	0.05	-0.21	-0.03
Anterior cingulate gyrus (ACgG) → PTSD symptom severity	-0.10	0.05	-0.19	-0.00
PTE → PTSD symptom severity	0.35	0.04	0.26	0.41

HTR3A, 5-hydroxytryptamine receptor 3A; NR3C1, nuclear receptor subfamily 3 group C member 1; SE, standard error; CI, confidence interval; PTE, potentially traumatic event; PTSD, posttraumatic stress disorder.

**Figure 3:**
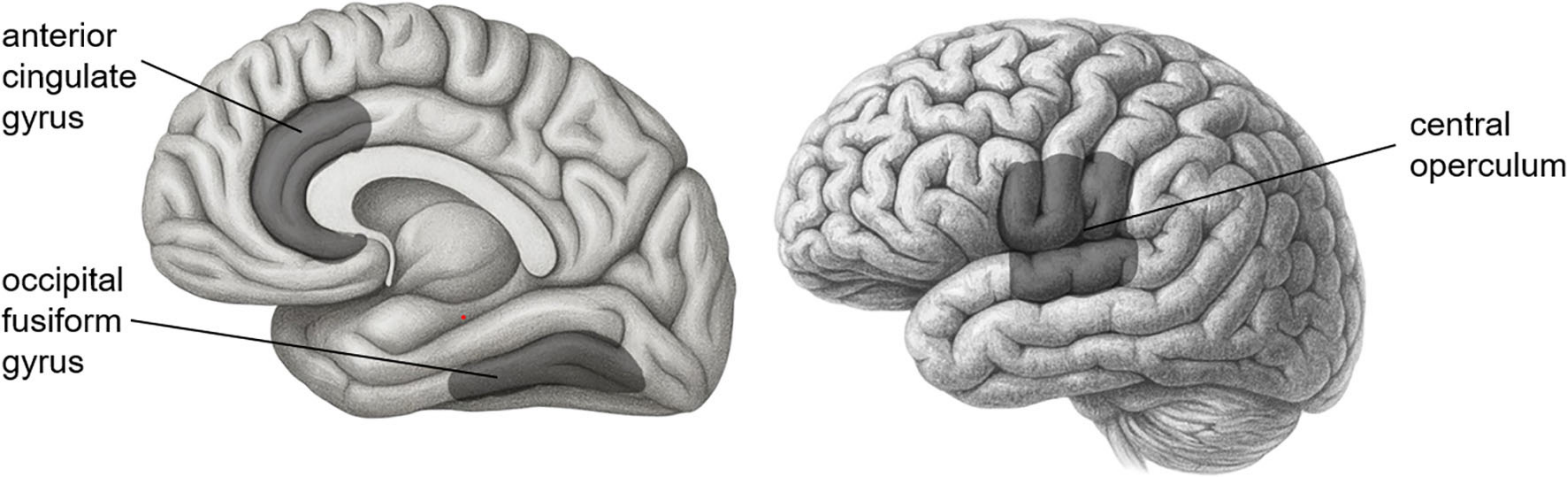
Three brain areas significantly mediated between genes and PTSD symptom severity.

For the relationships between brain ROIs and the severity of PTSD, the severity of PTSD turned out to be negatively associated with three brain ROIs—central operculum (*b* = -0.11, SE = 0.04, 95% CI = [-0.18, -0.00]), occipital fusiform gyrus (*b* = -0.13, SE = 0.05, 95% CI = [-0.21, -0.03]), and anterior cingulate gyrus (*b* = -0.10, SE = 0.05, 95% CI = [-0.19, -0.00])^1^, suggesting that people with smaller volume in those ROIs had more susceptible to the severity of PTSD symptoms. The PTEs also had a negative association with the severity of PTSD symptoms (*b* = 0.35, SE = 0.04, 95% CI = [0.26, 0.41]) even after the effects of ROIs and covariates were manipulated. It would imply that the traumatic experiences had a direct effect on the severity of PTSD symptoms even after adjusting the effects of covariates and decreasing the volume of ROIs. **Figure 3** shows three brain areas significantly mediated between genes and PTSD symptom severity.

In the pathways from genes to the three brain ROIs above, HTR3A had a negative influence on the anterior cingulate gyrus (*b* = -0.11, SE = 0.04, 95% CI = [-0.18, -0.03]), indicating that people with a more minor allele in HTR3A were likely to have a smaller anterior cingulate gyrus. However, the indirect effect of HTR3A on the severity of PTSD symptoms through the anterior cingulate gyrus was not statistically significant (*b* = 0.01, SE = 0.01, 95% CI = [-0.00, 0.02]). On the other hand, the effect of NR3C1 on the central operculum was positive (*b* = 0.09, SE = 0.03, 95% CI = [0.02, 0.15]), implying that people with a mutant allele in NR3C1 had a larger central operculum. Moreover, NR3C1 had an indirect effect on the severity of PTSD symptoms mediated through the central operculum (*b* = -0.01, SE = 0.01, 95% CI = [-0.02, -0.00]), which means that the mutation of the NR3C1 led people to be less vulnerable to PTSD, though enlarging their central operculum. None of the interaction effects of genes and PTE on the three ROIs were statistically significant. The ratios of the amount of explained variance for each ROI or the severity of PTSD to their total variances are in **Table 6** (average R^2^ = 0.25).

**Table 6:** R^2^ for all the dependent constructs, regions of interest (ROIs) and PTSD symptom severity.

Dependent constructs	R^2^	Dependent constructs	R^2^
Frontal Pole (FRP)	0.24	Angular Gyrus (AnG)	0.29
Superior Frontal Gyrus (SFG)	0.37	Postcentral Gyrus (MPoG, medial segment)	0.17
Middle Frontal Gyrus (MFG)	0.40	Precuneus (PCu)	0.32
Inferior Frontal Gyrus (IFG)	0.40	Superior Occipital Gyrus (SOG)	0.20
Precentral Gyrus (PrG)	0.41	Inferior Occipital Gyrus (IOG)	0.24
Superior Frontal Gyrus (MSFG; medial segment)	0.42	Middle Occipital Gyrus (MOG)	0.27
Supplementary Motor Cortex (SMC)	0.37	Occipital Pole	0.21
Medial Frontal Cortex (MFC)	0.26	Occipital Fusiform Gyrus (OFuG)	0.22
Gyrus Rectus (Gre)	0.21	Cuneus (Cun)	0.22
Subcallosal Area (SCA)	0.29	Calcarine Cortex (Calc)	0.23
Precentral Gyrus (MPrG; medial segment)	0.26	Lingual Gyrus (LiG)	0.22
Anterior Orbital Gyrus (AOrG)	0.27	Anterior cingulate gyrus (ACgG)	0.32
Medial Orbital Gyrus (MOrG)	0.28	Middle cingulate gyrus (MCgG)	0.35
Lateral Orbital Gyrus (LOrG)	0.35	Posterior cingulate gyrus (PCgG)	0.29
Posterior Orbital Gyrus (POrG)	0.31	Parahippocampal Gyrus (PHG)	0.18
Frontal Operculum (FO)	0.29	Entorhinal Area (Ent)	0.11
Central Operculum (CO)	0.38	Third Ventricle	0.27
Parietal Operculum (PO)	0.25	Fourth Ventricle	0.09
Anterior Insula (AIns)	0.25	Inferior Lateral Ventricles	0.06
Posterior Insula (PIns)	0.21	Lateral Ventricles	0.12
Temporal Pole (TMP)	0.14	Caudate	0.16
Superior Temporal Gyrus (STG)	0.26	Putamen	0.18
Middle Temporal Gyrus (MTG)	0.26	Thalamus	0.14
Inferior Temporal Gyrus (ITG)	0.17	Basal Forebrain	0.18
Planum Polare (PP)	0.30	Nucleus Accumbens	0.15
Transverse Temporal Gyrus (TTG)	0.30	Pallidum	0.08
Planum Temporal (PT)	0.28	Ventral Diencephalon	0.19
Fusiform Gyrus (FuG)	0.26	Amygdala	0.15
Postcentral Gyrus (PoG)	0.41	Hippocampus	0.18
Supramarginal Gyrus (SMG)	0.33	PTSD symptom severity	0.28
Superior Parietal Lobule (SPL)	0.28	Average	0.25

PTSD, posttraumatic stress disorder.

## Discussion

This study conducted a comprehensive examination of the path-analytic relationships between posttraumatic events, genes, their interactions, brain volumes, and PTSD symptom severity in a single modeling framework via IG-GSCA while controlling for the effects of possibly contaminating variables such as gender, age, and alcohol problems. In grounding these path‐analytic tests, we drew on the classic diathesis–stress model, which holds that genetic risk and environmental exposures (PTEs) jointly influence psychopathology. More recent extensions (71–73) further underscore how gene-environment interactions can shape brain structure and function. By embedding our IG‐GSCA interaction terms within this established G×E paradigm, we both structure our hypotheses and sharpen the interpretation of indirect pathways from genes, via brain structural changes, to PTSD symptom severity. Our major findings are threefold, as follows.

First, although the indirect effect of *HTR3A* on PTSD symptom severity via anterior cingulate volume was not statistically significant, we observed significant individual associations along the pathway. Specifically, the number of minor alleles (T) in the HTR3A gene polymorphism was negatively associated with the anterior cingulate volume, which was also negatively associated with the severity of PTSD symptoms. The HTR3A gene encodes subunit A of the type 3 receptor for serotonin, and the type 3 receptors are largely reported in the anterior cingulate cortex (74–76). While some studies suggest the relationship between the CC genotype (the wild type) of HTR3A and increased PTSD symptom severity (77), other opposite studies suggest the relationship between the number of minor alleles T of HTR3A and a higher pain score, which is related to higher distress (78). These contradictory results could be due to other contaminating variables, such as age, gender, ethnicity, and other covariates. In our Korean sample, controlling for age, gender, and alcohol-related problems, we found a significant negative relationship between the number of minor alleles and anterior cingulate volume. The decreased volume of the anterior cingulate, which involves fear-conditioning (79) and the hypothalamic-pituitary-adrenal (HPA) axis (80), is observed in people with PTSD (80–83). Therefore, while the mediation effect was not significant, the directionality of the observed associations is consistent with prior findings and may suggest a potential trend that warrants further investigation in larger samples.

Second, this study also revealed that the central operculum volume acted as a statistical mediator of the association between NR3C1 genotype and PTSD symptom severity. The number of minor alleles (G) in the NR3C1 gene polymorphism was positively associated with the volume of the central operculum, and the central operculum volume was negatively associated with the severity of PTSD symptoms. The NR3C1 gene codes for the glucocorticoid receptor gene, which is known as a key element involved in several steps of HPA axis modulation (84, 85). A previous study found that the presence of the G allele (the minor allele) in rs258747 significantly reduced the risk of PTSD (40). This is in line with our finding that the number of minor alleles in NR3C1 was associated with a decrease in PTSD symptom severity. The central operculum volume mediated this relation; the number of minor alleles was related to an increase in the operculum volume, which was negatively associated with PTSD symptom severity. A study suggested that the operculum region might be related to PTSD (86). From the Neuromorphometric atlas, the central operculum refers to the middle part of the operculum between the anterior limit of the insula and the posterior limit of the ventral bank of the ascending ramus of the posterior lateral sulcus above the circular sulcus of the insula. In other words, the central operculum is the region near the insula that is functionally connected to other regions such as the insula, anterior cingulate cortex, and thalamus. This functional network of brain regions is called the “cingulo-opercular network.” (87, 88) Since the cingulo-opercular network is known to be engaged in dealing with alertness, it could be related to the NR3C1 gene involved in HPA axis modulation, as in our result.

Third, in addition to the volume of the central operculum and anterior cingulate gyrus, the volume of the occipital fusiform gyrus showed significantly negative relations with the severity of PTSD as well. According to the Neuromorphometric Atlas used in this study, the region from the occipitotemporal and collateral sulci anterior to the antero-medial limit of the ventral bank of the parietooccipital sulcus is labeled the fusiform gyrus, and the region from these sulci that lies posterior to the antero-medial limit of the parietooccipital sulcus is labeled the occipital fusiform gyrus. The occipital fusiform gyrus is known to be associated with the recall of traumatic autobiographical episodes (89), and PTSD patients had a lower volume of occipital fusiform gyrus compared to healthy controls (90). Our result is in line with those of previous studies. However, none of the genes showed significant relationships with the occipital fusiform gyrus volume. Thus, the occipital fusiform gyrus would not mediate between gene or PTE expressions and PTSD symptom severity.

However, this study has some limitations. First, our IG-GSCA model explained only approximately 28% of the variance in PTSD symptom severity. This suggests that important variables may underlie the observed variation, such as other psychiatric comorbidities like anxiety, depression, bipolar disorder (91, 92). Future studies should consider incorporating these additional variables to improve the model’s explanatory power.

Second, the number of participants in our study was relatively small (N = 231) compared to the number of parameters in the structural model (number of paths = 1,384). Although IG-GSCA has been shown to recover parameters well even with small sample sizes (12), the statistical power to detect all true effects remains limited. For instance, only a few G×E interaction terms reached statistical significance (e.g., PTE × BDNF on MPrG). This pattern may reflect insufficient power to detect such interaction effects, rather than a lack of true underlying associations. Studies with larger sample sizes would be valuable for cross-validating our findings.

Third, our findings are insufficient to make strong causal claims for the hypothesized pathways between variables. While this study proposed a model in which brain structures mediate the effects of genes and an environmental factor on PTSD symptom—and found empirical support for this structure—it does not rule out alternative causal models. For example, PTSD symptom severity could plausibly influence brain structure rather than the reverse. Additionally, potential biases introduced by the regularization applied in this study may obscure true causal relationships, despite improving the model’s predictive generalizability. Therefore, researchers should be cautious in interpreting the observed associations as causal. Further studies that also model reverse pathways from PTSD symptom severity to genetic and neurobiological markers may help clarify the complex relationships among PTSD, genes, and brain structure.

Fourth, the participants of this study consisted of mixed samples of PTSD patients (n = 94) and healthy controls (n = 137). Although this mixing was intended to assemble a cohort broadly representative of the general population, it likely diluted group‐specific differences in brain structure and may have reduced power to detect some gene–environment–brain pathways that are unique to PTSD. Future studies should recruit larger, more homogeneous PTSD cohorts for primary analyses, and when mixed samples are unavoidable, conduct pre-specified subgroup or interaction tests (e.g., including diagnostic status as a moderator) to uncover disorder‐specific effects of genes and PTEs on brain structure.

Fifth, the PTEs score was assessed only for those who experienced traumatic events, excluding those they witnessed or learned about, because witnessing or learning scores seemed unreliable for some participants in this study. However, exposure through witnessing or learning can also contribute to PTSD symptomatology. Future studies should consider developing or utilizing more reliable assessment tools for indirect trauma exposure to examine their distinct effects on brain structure and symptom severity.

Lastly, we lacked detailed clinical-status data, such as standardized diagnostic procedures, time since PTSD onset, and treatment history, which may influence both brain structures and symptom severity. Future studies should include comprehensive clinical assessments to determine how diagnostic method, illness duration, and treatment history modulate the associations among genes, brain structure, and PTSD symptoms.

Nonetheless, this study is among the few that attempted to simultaneously examine the relationships among a wide range of variables related to PTSD symptom severity—genetic variants, an environmental exposure, gene-environment interactions, and brain ROIs, simultaneously—within a single integrative model. Our findings revealed potential mediating pathways from HTR3A polymorphism to the anterior cingulate gyrus volume to PTSD symptom severity and from NR3C1 polymorphism to the central operculum volume to PTSD symptom severity, though only the latter indirect effect reached statistical significance. In addition, we identified a direct association between the occipital fusiform gyrus with PTSD symptom severity. These results emphasized the importance of holistically investigating the biological pathways underlying PTSD symptoms. In particular, detecting stress-related brain changes may inform clinicians’ evaluation of both risk and compensatory adaptation across the PTSD trajectory. We believe that our findings could serve as a stepping stone for future studies aiming to elucidate the broader pathophysiological mechanisms of PTSD.

## Data Availability

The datasets presented in this article are not readily available because of the confidentiality regulations outlined in the Bioethics & Safety Act and the Personal Information Protection Act. Requests to access the datasets should be directed to the corresponding author.

## Footnotes

1 The estimate *b*, SE, and 95% CI denote the estimate of path coefficient, its standard error, and its 95% confidence interval, respectively.
